# The use of advanced tracking technologies for the analysis of mobility in Alzheimer's disease and related cognitive diseases

**DOI:** 10.1186/1471-2318-8-7

**Published:** 2008-03-26

**Authors:** Noam Shoval, Gail K Auslander, Tim Freytag, Ruth Landau, Frank Oswald, Ulrich Seidl, Hans-Werner Wahl, Shirli Werner, Jeremia Heinik

**Affiliations:** 1 Department of Geography, The Hebrew University of Jerusalem, Mount Scopus, Jerusalem, Israel; 2 Paul Baerwald School of Social Work and Social Welfare, The Hebrew University of Jerusalem, Mount Scopus, Jerusalem, Israel; 3 Department of Geography, University of Heidelberg, Berliner Strasse 48, Heidelberg, Germany; 4 Department of Psychological Aging Research, Institute of Psychology, University of Heidelberg, Bergheimer Strasse 20, Heidelberg, Germany; 5 Section of Geriatric Psychiatry, Department of General Psychiatry, University of Heidelberg, Voss-Str.4, Heidelberg, Germany; 6 Margoletz Psychogeriatric Center, Tel-Aviv Sourasky Medical Center and Sackler Faculty of Medicine, University of Tel-Aviv, 6 Weizman Street, Tel-Aviv, Israel

## Abstract

**Background:**

One of the more common behavioral manifestations of dementia-related disorders is severe problems with out-of-home mobility. Various efforts have been attempted to attain a better understanding of mobility behavior, but most studies are based on institutionalized patients and the assessment usually relies on reports of caregivers and institutional staff, using observational approaches, activity monitoring, or behavioral checklists. The current manuscript describes the research protocol of a project that measures mobility in Alzheimer's disease and related cognitive disorders in an innovative way, by taking advantage of advanced tracking technologies.

**Methods/design:**

Participants are 360 demented persons, mildly cognitively impaired persons, and unimpaired controls aged ≥ 65 in Israel and Germany. Data regarding space-time activities will be collected via a GPS tracking kit for a period of 4 weeks in 3 waves (one year apart) with the same participants (using a repeated measures design). Participants will be interviewed by use of a battery of instruments prior to and following GPS data collection. Further, a family member will complete a questionnaire both before and after data tracking.

Statistical analyses will strive to explain differences in mobility based on a wide range of socio-structural, clinical, affect-related and environmental variables. We will also assess the impact of the use of advanced tracking technology on the quality of life of dementia patients and care givers, as well as its potential as a diagnostic tool. Systematic assessment of ethical issues involved in the use of tracking technology will be an integral component of the project.

**Discussion:**

This project will be able to make a substantial contribution to basic as well as applied and clinical aspects in the area of mobility and cognitive impairment research. The innovative technologies applied in this study will allow for assessing a range of dimensions of out-of-home mobility, and provide better quality data.

## Background

As the world's population ages, dementia-related disorders are becoming more prevalent. Intellectual impairment and cognitive disease, such as that associated with many dementias, constitute a serious threat to the well-being of older adults. Estimates of the prevalence of dementia vary worldwide. A recent consensus study [1] indicates a world prevalence of 24.3 million in 2001, for a rate of 3.9% among those aged ≥ 60. The rate is expected to double every 20 years [1]. Prevalence among community-dwelling Jewish elders in Israel is estimated at 16.7% [2]. In Germany, about 7% of the population aged ≥ 65 are affected by dementia [3]. Rates increase with age, so that for Western Europe, prevalence is 1.5% of those aged 65–69, rising to 24.8% of those aged ≥ 85 [1] and over one third of those over age 90 in Germany [3]. Milder forms of cognitive impairment are even more prevalent. At least 14% of the young-old, i.e., those between 60 and 79 years of age are expected to develop mild cognitive impairment (MCI). The rate increases sharply with age with estimates reaching 98% in some studies [4].

One of the more common behavioral manifestations of dementia-related disorders is severe problems with out-of-home mobility which is critical for numerous aspects of older persons' quality of life [5]. Cognitive impairment and dementia are among the major threats to maintaining out-of-home functional capacity and preferred mobility patterns [6,7]. One behavioral manifestation of dementia is wandering which includes checking, trailing, aimless walking, walking directed towards an inappropriate purpose, excessive activity, and attempts to leave the house" [8] and is estimated in 20–25% of community-dwelling dementia patients [9,10].

There are various explanations of out-of-home mobility behavior. Person-environment interaction models contend that the processes and outcomes of aging are strongly dependent on the physical characteristics of the environment [11,12]. Subjective criteria, such as place attachment and cognitive-emotional bonding have also been found to play a major role in person-environment transactions as people age [13,14]. Recently studies have focused on the interaction of these problems with various environmental stimuli and previous behavior patterns, leading to a new theory – Need-driven Dementia-compromised Behavior [15-17].

Certain out-of-home mobility patterns may also put the elder at risk for abuse, mainly due to the means that caregivers employ to prevent the behavior, for example, by restraining or confining the elder. These efforts also lead to reduced mobility, which in turn may negatively impact autonomy, self-esteem and well-being [17,18]. Thus, mobility problems may pose a source of considerable distress to caregivers and families [19] which may lead to the institutionalization of the elder. One of the goals of the proposed study is to assess the extent to which caregiver stress and burden are associated with mobility problems.

Various measures exist for assessing the mobility of older adults. Most are based on assessments of the environment, subjective measures of safety and satisfaction, modes of transport and means of carrying out specific activities, e.g. shopping and leisure [20]. Most measures reported in the literature are indirect, and include classification by caregivers, observational approaches, activity monitoring, checklists, and selected items within scales of dementia behavior. To date, electronic tagging and initial use of GPS monitoring have only addressed boundary transgressions [21,22]. One of the goals of the project described here is to develop accurate measures of various dimensions of movement in time and space within the context of the general mobility patterns of older adults.

In recent years technological advances have sparked the development of a wide range of easily available tracking technologies that can be used to gather high-resolution spatial and temporal data for pedestrian research [23]. However, to date, research into human time-space activities, using tracking technologies has been largely limited to studies tracing of the spatial routes of motorized vehicles [24-26]. It is considerably more difficult to gather such data from pedestrians Only recently have tracking devices developed that will neither disrupt nor influence the actions of pedestrians, i.e. devices that are small, passive and reliable.

Currently, there are several digitally-based tracking methods that could be used to gather information on the spatial activity of pedestrians. ***The Global Positioning System ***is a series of satellites that orbit the earth broadcasting signals, which are picked up by a network of receivers. The position of each receiver is determined by triangulating the incoming data from at least four satellites. Any kind of obstruction will produce an inaccurate reading; and herein lays the GPS' principle drawback. Its main advantage, however, is that as a worldwide system, it virtually spans the globe.

***Land-Based Tracking Systems ***consist of a series of antenna stations [Radio Frequency (RF) detectors] which are distributed throughout a given local area [27]. The advantage of land-based technologies is that the end unit does not have to be exposed directly to the RF station. With no need for a direct line of sight between the system's antennas and the end unit, the latter can be placed in a bag or pocket, significantly reducing the burden on the subject. On the other hand, the data provided by these techniques is often less accurate than that supplied by GPS devices [23,28].

***Hybrid Systems ***combine elements from two or more systems in an effort to reap the benefits, while minimizing disadvantages of above technologies. The most common hybrid solution currently available is the Assisted GPS (AGPS). The advantages of this specific hybrid system are twofold: not only does it provide a much more accurate reading, particularly in enclosed areas, but it also eliminates the problem of having to incorporate large and unwieldy GPS receivers into today's trendy, miniature handsets (i.e. the system's end units), which can now be furnished with a partial, hence much smaller, GPS receiver [29].

**Figure 1:**
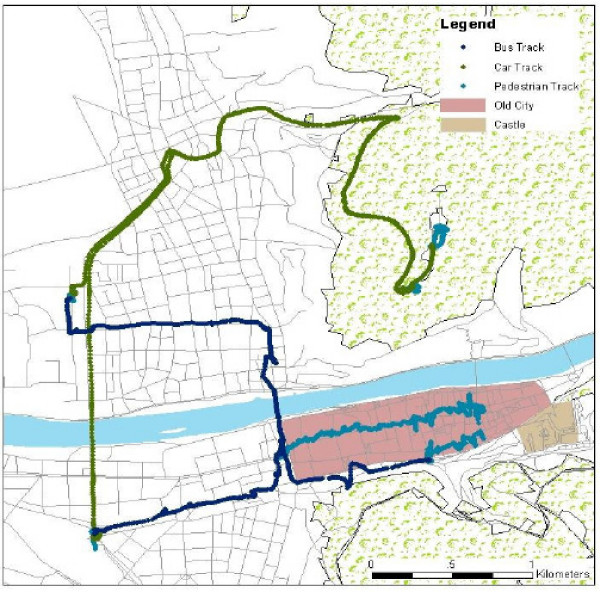
A visitor's activity in Heidelberg, as obtained by a GPS receiver every second.

The high resolution spatial and temporal data, obtained with GPS devices enables analysis of the characteristics of out-of-home mobility, such as the average pace of walking in different segments of the path, the time spent in different places, the total length of the trip. See for example figure 1 that represents the track of a visitor in Heidelberg, Germany, as obtained by a GPS receiver that was programmed to obtain a location every second.

These methods may be useful in both measuring out-of-home mobility in elderly dementia patients, and in intervening to manage that behavior. Managing that behavior, may, in turn, reduce the distress and burden of family caregivers and allow them to continue caring for the elder in the community for a longer period of time. Thus, a goal of this study is to assess whether the use of advanced tracking technologies reduces this stress, thereby improving both caregiver and elder's quality of life.

While the technology to allow for the electronic surveillance of elders' mobility patterns is becoming increasingly precise, sophisticated and non-intrusive, numerous ethical issues associated with its use have been raised [21,30-34]. Electronic tagging and tracking devices may be viewed as a way of creating a more secure environment for vulnerable persons who are at risk [21,31,35]. On the other hand they may also be viewed as a threat to human dignity and freedom [32,33]. Some critics argue that the use of these new technologies may deprive cognitively impaired elders of their privacy and necessary resources currently provided by informal or formal caregivers, and particularly reduce the human contact with their environment. However, the current social climate leans towards ever increasing liberal individualism thus enabling individuals to have more choice and freedom to decide about their care in the latter years of their lives [36]. It is important to balance the patient's safety and family well-being with the potential for abuse and threat to civil liberties [35]. Thus, an additional goal of the proposed project is to examine the ethical implications of electronic surveillance measures as perceived by healthy older people, those with various levels of cognitive impairment, family caregivers and professional care providers. This project is very timely given the recent increased support for electronic tagging of dementia sufferers by the UK's Alzheimer's Society and their call for more research into the possible merits of electronic tagging [37].

### Project aims

The current manuscript describes the research protocol of a project that addresses the feasibility and benefits of using advanced tracking technology to assess the out-of-home mobility of older adults, in Israel and Germany. The main goals and aims of the study are:

1. Collecting high resolution spatial and temporal data on the mobility of older adults which will allow to: (1) Test the suitability of the technology as a means of monitoring the spatial behavior of cognitively impaired patients; (2) Obtain data on the level of activity outside the home during the night and day; (3) Assess the acceptance and compliance with the tracking program; and (4) Conduct geo-statistical analyses to characterize the activities of each research subject in time and space.

2. Developing measures of mobility behavior through the use of advanced tracking technology including: (1) Identifying mobility patterns that are specific to people with MCI, mild dementia, and no cognitive impairment, as well as mobility patterns that are common to all. Among dementia patients, identifying mobility patterns associated with different stages of the disease; (2) Comparing findings in the two countries, in order to determine if there are symptoms or mobility behavior patterns that are environmentally and culturally linked as distinct from symptoms and behaviors that transcend these borders.

3. Assessing the extent to which elder and caregiver well-being are associated with mobility problems.

4. Assessing the potential of advanced tracking technologies to reduce stress and burden, thereby improving the quality of life of dementia patients and their families.

5. Examining the ethical implications of using advanced tracking technologies. We hope to be able to recommend a protocol for its use which safeguards of patients' rights while promoting well-being.

6. Assessing the potential contribution of advanced tracking technologies to the diagnosis of cognitive impairment and various types of dementia.

### Hypotheses of the current study include

1. Poorer cognitive functioning and higher frequency of mobility problems will be related to lower family well-being.

2. There will be an improvement in quality of life and level of burden among the MCI and dementia patients and the family members who used the equipment, and that improvement will be significantly greater than that of the healthy elders and of the MCI patients and families who did not receive the tracking intervention.

3. Patients who received the tracking intervention will have better quality of life and remain living in the community longer than similar patients who did not receive the tracking intervention.

**Figure 2:**
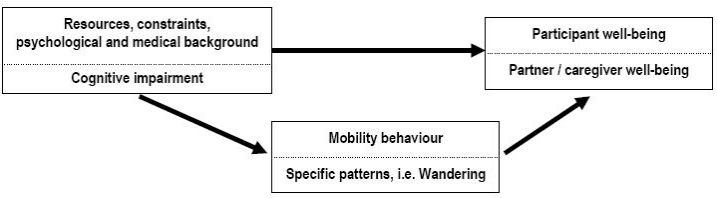
Conceptual model of the background and aims of the study.

Figure 2 summarizes the background and aims of the current study. As illustrated in the conceptual model, we posit that older persons' mobility is related to a number of factors including environment and personal resources and constraints, psychosocial and medical factors including, specifically, their cognitive state. We further posit that their well-being and that of their caregivers is substantially mediated by their mobility. It is also directly related to their resources, constraints, psychosocial and medical background. In addition, we propose that advanced tracking technologies have a dual role, both as a means of measuring mobility and as an intervention to improve well-being.

## Methods/design

### Participants

This is a bi-national study with participants and researchers from both Israel and Germany. Participants will be residents of the Greater Tel Aviv metropolitan area in Israel and the Rhine-Neckar metropolitan region in Germany. Participants are a core sample of 360 people aged ≥ 65, with 180 in each country (600/300 to allow for attrition). These will include equal numbers of men and women, and people with MCI, mild dementia, and no cognitive impairment (see table 1).

**Table 1:** Overview of the sample plan

	Core sample – Wave 1					
Sample	Healthy		Mild Cognitive Impairment		Persons with mild dementia	
	Women	Men	Women	Men	Women	Men
Age	65+	65+	65+	65+	65+	65+

Germany	50	50	50	50	50	50
Israel	50	50	50	50	50	50

**Total**	**200**		**200**		**200**	

Assignment to the three sample groups is based on the clinical diagnoses using DSM-4 operational criteria for dementia (Alzheimer's type and mixed) [38]. MCI criteria is based on Petersen et al [39] and Winblad et al [40] criteria and employs CAMCOG-R [41] in Israel and CERAD [42] in Germany. The following instruments are administered: MMSE [43], CDT-MIA [44], CAMCOG-R [41], CERAD [42], TMT A+B [45], GDS [46], NPI [47], AES-C [48] and CDR [49]. The lowest MMSE score accepted in the study is 21. Exclusion criteria are: severe motor and motility disorders (e.g. Parkinson disease), sensory impairment, certain medical problems, a history of substance abuse, major psychiatric illness or no fluent language skills. Elders residing in institutions at the start of the study will also be excluded. Only elders with a caregiver, potential caregiver or significant other living in close proximity will be included.

In order to assess the impact of the tracking technology on patients and their family members, we will also recruit a control group of MCI and mild dementia patients who will not be provided with a GPS tracking kit.

Further, 12 persons in each of two focus groups (family caregivers and professionals) and fifty persons in each of four groups (older people with no cognitive impairment; persons with MCI or mild dementia; family caregivers of MCI and dementia patients in the community; and various professional care providers) will be recruited for the ethics study.

### Sample size calculations

The sample size was derived from a power analysis, considering a 2 [gender] × 3 [competence group] analysis of variance as "typical" non-descriptive analysis to be computed for relevant dependent variables of the study. To determine the required sample size, we followed the approach proposed by Cohen [50], see in particular formula 8.4.4, p. 396) for equal cell sizes. To detect "medium" main or interaction effects (f ≥ .25) with sufficient power = 0.8 in F-tests with α = .05 significance level, a cell sizes of n = 27 is needed, which we rounded to n = 30 to be "on the safe side", hence the total sample size intended was N = 180. Expecting attrition rates of 20% over the course of the study (i.e., from wave 1 to 2 and from wave 2 to 3), total N = 281 would be required to assure sufficient power also in cross-sectional analyses of data from the wave 3, we once again rounded this number to the desired sampling goal of N = 300 for each research site.

### Recruitment

Impaired participants will be recruited from The Margoletz Psychogeriatric Center, Tel-Aviv Sourasky Medical Center in Israel and the Department of Geriatric Psychiatry, University of Heidelberg in Germany. Non-impaired participants will be recruited by convenience sampling from a variety of sources (senior centers, friends and family of patients) in Israel and by random sampling techniques from the regional official registers in Heidelberg, Mannheim, and Ludwigshafen in Germany. Non-impaired participants will also be screened using the above mentioned tests. Those who show indications of MCI or dementia will not be included in the non-impaired sample and will be offered referral for a full diagnostic work-up, through their health care provider.

### Ethics

Ethic approval for the project was obtained from the Institutional Review Board ("Helsinki Committee") of the Israeli Ministry of Health and the Ethic Board Review of the University of Heidelberg.

### Procedures and data collection instruments

The research will span a period of five years. Data regarding space-time activities will be collected from each subject in 3 waves, one year apart, using a repeated measures design. This will allow us to identify changes in cognitive status and mobility patterns over time, particularly in the MCI and mildly demented groups, so that some participants may actually be moderately demented (or worse) by Wave 3.

During the first year of the study, we carryied out extensive pre-tests of the data collection instruments and procedures on a sample of 30 healthy elders, elders with MCI and mild dementia in each country. In years 2–4 of the project we will collect data from the core sample.

Great effort has been made so that instruments distributed in both countries will follow identical order as much as possible. However, there are some minor difference between the two countries which are related to cultural variation as well as technical issues in the two countries.

Data will be collected in three interviews, as described below. Potential participants of the memory clinics will be sent a letter containing information on the research and inviting them to participate in the study. Similar information will be given to potential non-impaired participants prior to enrollment in the study.

### First interview

Participants who agree to participate in the study will be invited to a first meeting either within the memory clinic in Israel and within the memory clinic or the Department of Psychology of Heidleberg University in Germany. In this meeting a trained psychologist will explain the study's goals and procedures. The participant and their family member will be able to ask questions and will then sign the consent form. At this time the interviewer will collect demographic background information (sex, year of birth, marital status) and necessary data for cognitive assessment (using instruments described above), chronic diseases and medications.

### Second interview

Following the meeting at the memory clinic, a second interview with the participant and their family member will be scheduled to take place at the participant's home. At this time, both the participant and the caregiver will complete questionnaires. The participant will be interviewed via a battery of questionnaires (approximately one hour), while the family member will complete the questionnaires independently (the family member will be able to ask the interviewer clarification questions if needed).

### Participant interview

(1) Sample description – Background information not included in the first interview will be asked at this time: e.g. people living in same household, pets, country of birth, mother tongue and education.

(2) Basic housing conditions – (The Housing Enabler, long version/short version; [51]). This section will include information on housing conditions (house/apartment, floor), household composition, housing amenities (e.g., size, no. of rooms, heating), housing tenure, duration of living in town and house, how secure the participant feels in their neighbourhood and the participant's economic situation (in Germany the economic situation will be asked at the third interview).

(3) The Geriatric Depression Scale[46] – this instrument is intended to measure depression specifically with the older population. The short form includes 15 items to which participants are asked to respond by answering yes or no in reference to how they felt over the past week. The GDS may be used with healthy, medically ill and mild to moderately cognitively impaired older adults. In Germany, in case of healthy participant, this instrument will be utilized for the first time within this interview while in case of non-healthy participant the GDS score will be collected from the clinic file. In Israel it will be conducted within the first interview at the memory clinic.

(4) Subjective Health Status – This includes 5 items on perceived health, mobility, vision and hearing [52]. Participants are also interviewed for functional health (SF-36, [53]) including items that assess: Physical functioning, physical role-related functioning, pain, social functioning and emotional role-related functioning.

(5) List of important activities and most important outdoor places – From an extensive list of activities and services, the participant is asked to indicate in which of these they take part, whether they engage in activities by themselves or while accompanied by another person, the frequency of the activity and its location (street address or junction). These locations will be mapped by geographers and checked after tracking period. The participant is also asked which of the activities and places are most important to them.

(6) An open-ended question allows the participant to note other factors that effect their outdoor mobility.

(7) Perceived functional independence[54] is a single item in which the participant rates how they perceive their independence in activities of daily living.

(8) House and Environment This section examines indoor-outdoor motivation (ENABLE-AGE), a personal behavioural tendency that varies according to biographical experiences and preferences that have developed across the life span [55]. The motivation-oriented attitude is assessed with a global rating that addresses the participant's ideal position between the extremes of staying at home versus being outside as much as possible. Indoor and outdoor place attachment – (ENABLE-AGE) addresses cognitive and emotional bonding to the own home [55]. This is assessed with two 11-point rating scales from 0 ("not at all attached") to 10 ("fully attached"). Most favourite outdoor places – this open question asks participants to recall their favourite place either currently or from the past.

(9) Environmental Mastery (Ryff, 1989) Since psychological well-being is multi-dimensional it is important to measure various facets of this concept. The Ryff-scales on psychological well-being [56] represents aspects such as purpose in life, or from an environmental point of view, autonomy and environmental mastery. The scale is based on nine items on a 5-point rating scale. A person with high scores has a sense of mastery and competence in managing the environment; controls complex array of external activities; makes effective use of surrounding opportunities; able to choose or create contexts suitable to personal needs and values. A person with low scores has difficulty managing everyday affairs; feels unable to change or improve surrounding context; is unaware of surrounding opportunities; lacks sense of control over external world.

(10) Social Network and Social Support – Assessment of social network via frequency of contact with family, friends and neighbours according to a seven-item scale developed by the Israeli Central Bureau of Statistics [57]. A single item asks about the number of close friends and family members. Further, a 6-item social support questionnaire [58] asks respondents to rate the availability of various types of supports.

(11) Emotional well-being (affect) (Positive And Negative Affect Schedule; PANAS)[59]. This instrument is used to assess emotional well-being as part of healthy ageing. It provides a score for emotional balance (i.e., the difference between the frequencies of positive and negative affect) as well as an independent score for negative and positive affect.

(12) Two open questions allow participants to add any additional important information that was not otherwise elicited in the survey.

(13) Life satisfaction – Single-item rating on life satisfaction (ENABLE-AGE).

(14) Interviewer comments – the interviewer is asked to complete several questions following the interview regarding participant's understanding and reliability of their answers as well as difficulties that arose.

### Caregiver questionnaire

While the participant is being interviewed, caregivers will be asked to complete a battery of questionnaires. Some instruments are identical to those completed by the participant and discussed above: basic housing conditions and background information; perceived health, mobility, vision and hearing; SF-36; life satisfaction; social network and social support and emotional well-being – affect (PANAS). Additional instruments completed solely by the caregiver include:

(1) The caregiver is asked whether the participant tends to go out by themselves or accompanied by someone else.

(2) Autonomy allowance – This section includes two questions. The first question asks "Sometimes, individuals suffering from memory impairments feel the need to wander around or to go outside without realizing that this may result in unpleasant consequences. Have you experienced this?" This question is answered by "yes" or "no". For a "yes" response, the caregiver is asked to elaborate on how they deal with such situations.

(3) Apathy Evaluation Scale (AES, Marin, 1991[60]) – This scale examines apathy in the participant as perceived by the caregiver. Apathy is defined as "lack of motivation not attributable to diminished level of consciousness, cognitive impairment, or emotional distress." The scale is composed of 18 items rated on the degree to which each statement is true of the participants. Statements refer to either cognitive, behaviour, emotional or other.

(4) Center for Epidemiologic Studies Depression Scale (CES-D) (Radloff, 1977[61]) – The scale is a short, self-reporting scale intended to measure depression in the general population. The scale is composed of 20 items rated for their frequency of occurrence in the previous week. The answers range from "rarely or none of the time (less than 1 day)", "some of or a little of the time (1–2 day)", "occasionally or a moderate amount of the time (3–4 days)" and "most or all of the time (5–7 day)". The first option is rated as 0 while the last category is rated as 3. Thus, summary scores can range from 0 to 60 with higher scores indication more depression symptomatology.

(5) Zarit Burden Interview Short Form (12-Items)[62] – Assesses the stress experienced by family caregivers. Caregivers are asked to indicate how often they have felt each of 12 items. A different in the use of this scale will occur between the two countries in relation to the healthy sample. While in Israel all caregivers of participants in the healthy sample will complete the Burden Scale, this scale will not be mandatory for caregivers of healthy participants in the German sample.

### Intervention

**Figure 3:**
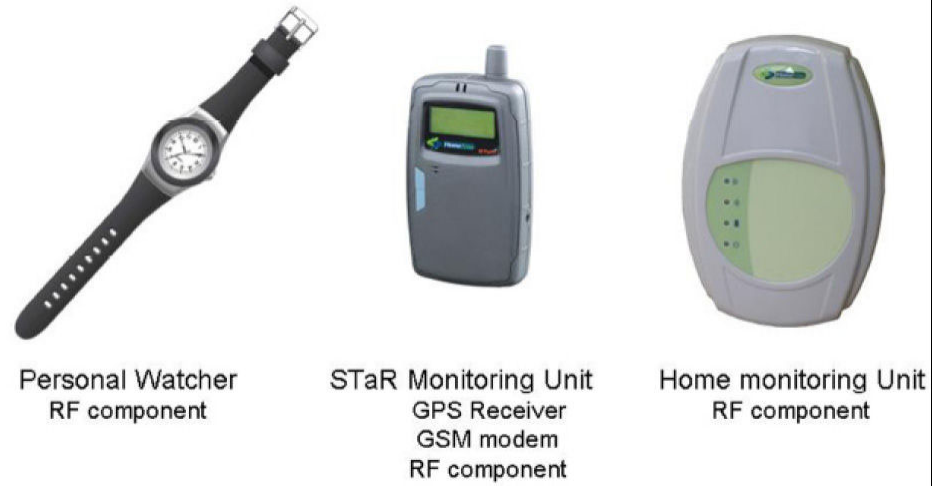
Elements of the location kit to be used in the project.

Following the second interview, the participant will receive a GPS tracking kit. At this time, a full explanation of the use of the kit will be given. The participant can choose how to carry the kit, within a belly pouch, on the shoulder (like a shoulder-bag), or in any other way that is convenient to the participant. The participant will take the GPS kit with him/her everywhere during the whole day for a period of 4 weeks. The GPS tracking kit that will be used (see figure 3 for the elements of the kit) consists of a GPS receiver with a GSM modem, an RF transmitter contained in a wrist-watch and a monitoring unit located in the home that enables researchers to know whenever the tracked person leaves home. The waterproof RF transmitter (no need to take off during shower, for example) will allow researchers to know whether or not research subjects leave home with the GPS device and if they are carrying the device or not at a given moment. This feature is critical to assessing the validity level of tracking during the relatively long period of tracking.

The GPS is programmed to obtain locations every 10 seconds when the tracked person is outside the home. The data collected in Israel and in Germany are sent by GPRS protocol to a control unit at the Hebrew University of Jerusalem where it is stored on the project's server. Family members of patients in the study group will be able to log onto the project web site to locate their family member in real time.

During the four weeks of tracking, interviewers carry out weekly phone conversations with the participants in order to inquire on the elders' well-being and possible difficulties in using the GPS kit. Further, participants will be asked to keep a daily log of their activities including their out-of-home trips. This will allow for additional validation of the tracking data.

### Third interview

Following 4 weeks of mobility tracking, the participant and caregiver will meet with an interviewer for a third interview. At this time the participant will be interviewed via: Perceived health, mobility, vision and hearing; objective functional health (SF-36); life satisfaction; emotional well-being (PANAS); and the Geriatric Depression Scale (GDS). Further, fear of falling will be assessed using the Falls Efficacy Scale – International Version (FES-I, [63]). The FES-I assesses confidence in performing a range of both easy and difficult physical and social activities of daily living without falling. Finally, the satisfaction of the participant with the use of the GPS kit will be examined by employing 8 questions on satisfaction derived from the Quebec User Evaluation of Satisfaction with Assistive Technology (QUEST 2.0,[64]).

At the end of the third interview the participant will be asked to identify nodes which the geography team was not able to identify. Nodes will be defined as places in which the participant stayed for a period of 5 minutes or more. With the help of the daily diary the interviewer will attempt to understand the location and purpose of each of the nodes during the past 4 weeks.

Caregivers will also be asked to complete a battery of questionnaires at this time. Besides the background information, all of the questionnaires from the previous meeting will be completed for a second time. Further, caregivers will also be asked to rate their satisfaction with the GPS kit using the QUEST.

### Additional instruments

For the purposes of comparing mobility behavior as assessed through the new GPS technology versus the perceived mobility behavior as reported by a caregiver, a previously validated survey instrument will be used. Algase et al. [16] version 2 is a 33-item questionnaire measuring frequency, pattern or quality, boundary transgression deficits in navigation or way-finding and temporal distribution of wandering behaviors. This questionnaire will be computed during the first interview with a sub-sample of the caregivers.

For the purposes of examining the ethical implications of using advanced tracking technologies with this specific population, the focus groups will meet twice: at the beginning of the research study and 2 years later. Participants will be asked regarding their views on the use of electronic surveillance devices for cognitively impaired persons who wander. Results from the focus groups will allow for the construction of the ethics questionnaire which will be given to the 4 groups of individuals described above in the participant section.

### Statistical analyses

This section describes some of the statistical analyses that will be conducted in this study. First, in order to analyze the huge amounts of mobility data that will be produced by the location kits, a new method for sequence alignment analysis of spatial activity will be implemented in order to create individual typologies for each research subject's activities and composite typologies of all the research subjects together. The need to use new methods to analyze the time-space factor arises from the fact that the different existing approaches for analyzing time-space activities in geography, including the traditional approach of prisms of possible time-space activities [65] and the newer methods based on GIS [66,67], are all incapable of generalizing the time-space patterns of different individuals into a "typical" time-space pattern based on analytical tools, while keeping the sequential elements as well. In this research we intend to use a recent modification (ClustalTXY 0_2) of the ClustalG software, that has been adapted for use in social science studies a decade ago [68] and recently in geography as well [69]. This is a modification of ClustalX that is widely used in molecular biology that is based on Sankof and Kruskal's [70] algorithm, since the early 1990's for comparing sequences of amino acids.

Second, we will compare the well-being of family members with the elders' mobility scores and cognitive functioning. We will also examine factors related to these outcomes in caregivers, such as background variables, social support, well-being, as well as patient characteristics. Further, we will evaluate the use of the tracking devices, with particular attention to patient and caregiver characteristics that are related to compliance and cooperation.

Further, we will examine differences in levels of burden and well-being between the study group, those who received the GPS kit and their family member, and the control group, those who did not receive the GPS tracking kit and their family members. Additionally differences in the different measures between second (prior to intervention) and the third interviews (following 4 weeks of intervention) will be examined. Further, we will also examine changes in living arrangements (hospitalization, assisted living, long-term care) and compare such changes between the study group and the control group.

Finally, statistical analyses will also be aimed at examining the potential use of tracking technology in diagnosis of dementia and in predicting the trajectory of the disease in previously diagnosed individuals. We will use data regarding spatial activity outside the home, as well as the percent of time spent outside the home, average distance of walking by foot per week, participants' ability to charge the location kits, and the number of times the research subject left the home without the location kit in relation to level of cognitive impairment as well as change in cognitive impairment over time. We will assess the (a) concurrent validity; (b) discriminative power, and (c) predictive value of these measures.

## Discussion

By achieving its goals, the project will be able to make a substantial contribution to basic as well as applied and clinical gaps in the area of mobility and cognitive impairment research. The project is novel and innovative in several respects. First, it utilizes and applies, for the first time in a systematic large-scale research project, continually evolving tracking technologies to a growing medical and psychosocial problem among older adults. Those technologies will allow for assessing more dimensions of the behavior, and provide better quality data, i.e., higher resolution, both in time and in space. Second, the project involves the collaboration between diverse disciplines – geography, medicine, social work, gerontology, ethics and psychology – in order to conduct a comprehensive examination of the issues and outcomes involved. Third, as opposed to other studies that seek to find *ad hoc *solutions to the ethical issues involved in the research, the current study includes an in-depth examination of the ethical issues involved in both the research and the intervention itself, in the hopes of pre-empting the imposition of technology on patients before acceptable protocols and limits are determined. Fourth, the cross-national comparison in this project takes advantage of the expertise of a diverse group of researchers in each country, and will enable us to evaluate whether the mobility patterns observed are culturally-linked or consistent across cultures.

## List of abbreviations

AES-C = Apathy Evaluation Scale, Clinical Version; CAMCOG-R = Cambridge Cognitive Examination – Revised; CDR= Clinical Dementia Rating; CDT-MIA = Clock Drawing Test – Modified and Integrated Approach; CERAD = Consortium to Establish a Registry for Alzheimer's Disease; DSM = Diagnostic and Statistical Manual of Mental Disorders; GDS= Geriatric Depression scale; GPS = Global Positioning System; MCI = Mild Cognitive Impairment; MMSE = Mini Mental State Examination; NPI = Neuropsychiatric Inventory; TMT = Trail Making Test

## Competing interests

The author(s) declare that they have no competing interests.

## Authors' contributions

Each of the authors contributed relevant material based on accepted practice and knowledge in their respective countries and disciplines. Integration of the material was done by GKA and NS. All authors took part in preparation of the manuscript and provided critical intellectual interpretation and manuscript revision. All authors read and approved the final manuscript which was written by SW.

## Pre-publication history

The pre-publication history for this paper can be accessed here:


